# Investigation of new dyes for chromovitrectomy: preclinical biocompatibility of trisodium, orangell and methyl violet

**DOI:** 10.1186/s40942-015-0003-x

**Published:** 2015-04-15

**Authors:** Emmerson Badaro, Rodrigo A Souza-Lima, Eduardo A Novais, Mauricio Maia, Flávio Hirai, Carsten H Meyer, Michel Eid Farah, Eduardo B Rodrigues

**Affiliations:** 1 Department of Ophthalmology, Federal University of São Paulo, 821 Botucatu Street, 1st floor, Sao Paulo, CEP Brazil; 2 Department of Ophthalmology, University of Bonn, Bonn, Germany

**Keywords:** Chromovitrectomy, Vitretomy, Trisodium, Orangell, Methyl violet, Retina, Toxicity, Eletroretinography, ERG

## Abstract

**Purpose:**

To investigate the retinal toxicity by electroretinography (ERG), clinical examination and histology after intravitreal injection of biological stains in two concentrations: Trisodium (0.50 g/L and 1.00 g/L), Orangell (0.25 g/L and 1.00 g/L) and Methyl Violet (0.50 g/L and 1.00 g/L).

**Methods:**

Eighteen New-Zealand albinos rabbits were assigned in six groups (n = 3 in each group). The animals in group 1 received Trisodium in the dose of 0.50 g/L and group 2 received 1.00 g/L; Group 3 received Orangell in the dose of 0.25 g/L and group 4 received 1.00 g/L; Group 5 received Methyl Violet in the dose of 1.00 g/L and group 6 received 0.50 g/L. A volume of 0.05 mL of dye was injected in the right eyes, whereas the left eyes received the same volume of balanced salt solution (BSS) as control. ERG recordings and clinical examination were performed at baseline and seven days after intravitreal injection. The ERG responses at one week after injection were compared with baseline levels. A decrease in the post-injection amplitude of more than 50% was considered remarkable. After the 7-day follow-up, rabbits were euthanized and eye enucleated for light microscopy (LM) histological evaluation.

**Results:**

At clinical examination by indirect ophthalmoscopy seven days after dye injection, all eyes were negative for cataract, hemorrhage, retinal detachment, and intraocular opacities. Amplitude analysis of maximum scotopic b-wave showed no significant reduction in either dye injected or control eyes. Neither dye nor BSS caused significant retinal alteration on LM at doses tested.

**Conclusions:**

Trisodium, Orangell and Methyl Violet can be applied in future studies in order to prove the capacity to stain preretinal tissues and vitreous without toxicity. The three dyes did not induce significant ERG amplitude reduction or LM alterations in this preliminary experimental research. Trisodium, Orangell and Methyl Violet may be potentially useful vital dyes for ocular surgery, and deserve further investigation.

## Background

Pars plana vitrectomy enables treatment of important diseases of the retina such as diabetic retinopathy, macular hole and retinal detachment. The removal of internal limiting membrane (ILM) and preretinal membranes, such as those proliferative vitreoretinopathy, may be a technically difficult surgical step, as these are thin and semi-transparent tissues. Chromovitrectomy refers to the use of dyes to stain preretinal membranes and vitreous, facilitating their intraoperative visualization and removal, thus meaning an advance in surgical technique and consequent improvement of surgical outcomes [1].

Initially, indocyanine green (ICG) dye was shown to stain avidly the internal limiting membrane (ILM) [2]. However, numerous reports have raised concerns over retinal toxicity after intravitreous ICG injection [3-5], and subsequently, different agents arose as newer alternative for vital dyes^,^ such as Trypan Blue (TB), Brilliant Blue (BriB) and Triamcinolone Acetonide (TA) [6-8]. While TB has good staining capacity for epiretinal membranes (ERM), BB stains ILM elements, and TA deposits well in the acellular vitreous [9-11]. BriB showed good biocompatibility for chromovitrectomy and emerged as an alternative to ICG in chromovitrectomy [12,13]. However, the safest and best dye with high affinity for ILM and ERM is yet to be determined.

The development of novel vital dyes is under intense investigation and several dyes have been tested in recent years in search of the ideal dye as the inappropriate use of these substances can lead to toxicity and considerable functional loss.

Controversy about the optimal dye increases. An ideal vital dye for chromovitrectomy must be capable of selectively staining the internal limiting membrane and epiretinal membrane, leaving the retina unstained, and providing adequate colour difference between the ERM/ILM and the retina. Providing adequate color difference between the ILM/ERM stained and normal retina. Other favorable characteristics are rapid elimination from the vitreous cavity, photochemical stability, solubility in BSS, lack of toxicity, and proper light absorption profile [14].

For this study, a total of three chemical biological stains were selected: Methyl Violet (MV); Trisodium (TR); and Orangell (OR). The aim of this paper was to investigate in vivo the retinal biocompatibility of these three dyes injected intravitreally into rabbits’ eyes with clinical examination, histology with light microscopy (LM) and electroretinography (ERG).

## Methods

### Dye solutions, pH, osmolarity, and light absorbing properties

A total of 5 and 10 mg of TR and MV (Merck, Darmstadt, Germany), 2,5 and 10 mg of OR (Merck, Darmstadt, Germany), in the powder form, were weighed with an analytical balance (Mettler-Toledo Inc., Columbus) and suspended in 10 mL of solvent, glucose 5%, water or BSS, to obtain a concentration of 0.05 g/L and 0.10 g/L for TR and MV, 0.025 g/L and 0.10 g/L OR. The mixtures were shaken for 5 minutes and sonicated (Unique Ind., Idaiatuba, Brazil) to obtain a complete solution. The pH was measured with a pH meter (Quimis Inc., Diadema, Brazil), whereas osmolarity was determined with an osmometer (Advanced Instruments Inc., Norwood). Vital dyes with pH and osmolarity closest to 7.0 and 290 mOsm were selected for the experiments. Spectral absorption was measured with a spectrometer between 200 and 1,000 nm immediately after preparation (Spectronic Genesys 5, Milton Roy).

### Animals and surgical technique for Intravitreal injection of vital dyes in Rabbits’ eyes

A total of 18 male rabbits, 1.8 to 2.5 kg, were used in accordance with the guidelines set forth by the ARVO (Association for Research in Vision and Ophthalmology) Statement for the Use of Animals in Ophthalmic and Vision Research and with the university guidelines regarding animal experimentation in ophthalmic and vision research. The animals were fed standard laboratory food and allowed free access to water in an air-conditioned room with a 12-hour light–dark cycle.

After the preparation of the drug solutions, 0.1 mL of 0.05 g/L or 0.10 g/L MV, 0.05 g/L or 0.10 g/L TR, 0.025 g/L or 0.10 g/L OR was injected into the vitreous cavity of the right eye (OD), and 0.1 mL of BSS (290 mOsm) into the left eye (OS) was used as control. After the placement of a lid speculum, a drop of topical povidone 5% iodine was instilled followed by BSS washout. The vitreous cavity was entered through the superotemporal sclera 2 mm posterior to the limbus using a 27-gauge needle connected to a 1-mL syringe containing dye or control solution. One drop of antibiotic–steroid solution was then instilled.

### Clinical evaluation with fundoscopy and biomicroscopy

Indirect ophthalmoscopy and slit-lamp biomicroscopy were performed before the injection, immediately after and 7 days after. Indirect ophthalmoscopy (Keeler Instruments, Broomall) and slit-lamp biomicroscopy were performed for detection of corneal clarity, appearance of the lens and retina, conjunctival reaction, and vitreous hazing.

### In vivo functional retinal toxicity by ERG

Electroretinography recordings were taken before the intravitreal injection (baseline) and 7 days after intravitreal injection. The rabbits were anesthetized with intramuscular injection of a solution containing 1 mL of ketamine (50 mg/mL) and 0.4 mL of xylazine (10 mg/mL). The pupils were dilated (tropicamide 1%) and the cornea was anesthetized with 1% proparacaine drops. The rabbits were placed on a heating pad during the experiment, unipolar contact lenses with ERG jet electrodes (Universe SA, La Chaux-de-Fonds, Switzerland) were put on both corneas with 2% methylcellulose (Ophthalmos, São Paulo, Brazil). A reference electrode filled with electrolytic gel was placed at the temporal cantus of the eyelid, whereas the ground electrode also filled with gel, was placed on the ear lobe. They were then presented in a Ganzfeld stimulator of the Veris system (Electro-Diagnostic Imaging Inc., San Mateo). After 30 minutes of dark adaptation, the procedure was performed and peak-to-peak amplitude analysis in scotopic maximal responses were analyzed. The responses one week after injection were compared with baseline levels. A decrease in the post-injection response of >50% in scotopic maximal b-wave was considered significant.

### In vivo morphologic retinal toxicity and cell counting with light microscopy

After the 7-day follow-up, rabbits were euthanized with intravenous injection of 120 mg/kg sodium pentobarbital and the eyes were enucleated for light microscopy (LM) histological evaluation. All eyes were sectioned in half and fixed at 4°C in a mixture of 2.5% glutaraldehyde and 4% paraformaldehyde in 0.1 moles/L phosphate buffer at pH 7.4. The specimens were stained en bloc with lead citrate, washed three times in 0.1moles/L phosphate buffer, and dehydrated with ethyl alcohol. Then, the specimens were embedded and stained with 1% toluidine blue and examined with an Optiphot-2TM (Nikon, Tokyo, Japan) for LM. Samples were collected from two different areas in all the dye-injected eyes in three serial sections: 500 μm inferior to the optic nerve and 4 mm from the optic nerve at the temporal-inferior quadrant. A horizontal diameter of the retinal surface of 1,100 μm was used for detailed analysis of retinal toxicity.

For histological evaluation of the degree of cellular injury, cellular abnormalities such as vacuolization, edema, or necrosis were described as “no” changes, “focal” abnormalities with less than 12 cells/ structures damaged, or “diffuse” changes with more than 12 cells/structures damaged [13,15]. Eyes were analyzed by two masked examiners in regard to dye, concentration and exposure to avoid bias. The inner and outer nuclear layers were counted for number of cells with the software program NIH ImageJ (NIH, Bethesda) within the 1,100 μm horizontal surface. The eyes of the dye group were compared with the BSS-control group; statistical analysis was performed using analysis of variance (ANOVA) followed by the Newman–Keuls test with a specific software program (GraphPad Software Inc., San Diego).

## Results

### Dye solutions, osmolarity measurements, and light absorbing properties

Mixed and sonicated dye preparations were used as complete solutions for injections. MV, TR and OR dyes were chosen in the BSS solvent. The osmolarity of selected dyes for experiments ranged from 267 mOsm to 315 mOsm, whereas pH values were within 6.85 and 7.23. Most vital dyes showed substantial absorption between 450 and 650 nm, whereas no agent demonstrated spectral absorption above 700 nm (Table 1).

**Table 1:** **Dye solutions, osmolarity measurements, and light absorbing properties of each dye tested**

**Dye solutions**	**Osmolarity measurements (mOsm)**	**pH values**
Trisodium 0.50 g/L	287	6.94
Trisodium 1.00 g/L	302	7.05
Orangell 0.25 g/L	267	6.85
Orangell 1.00 g/L	285	6.97
Methyl Violet 0.50 g/L	298	7.02
Methyl Violet 1.00 g/L	315	7.23

### Clinical evaluation with fundoscopy and biomicroscopy

After intravitreal injection, MV, TR and OR appeared as a floating purple, green or orange mass in the vitreous. At clinical examination by indirect ophthalmoscopy and slit-lamp biomicroscopy, seven days after dye injection, all eyes were negative for cataract, hemorrhage, retinal detachment, intraocular opacities, corneal desepitelization, infiltration and conjunctival reaction. No other adverse effects were observed in our study.

### Morphologic retinal toxicity with light microscopy

**Figure 1:**
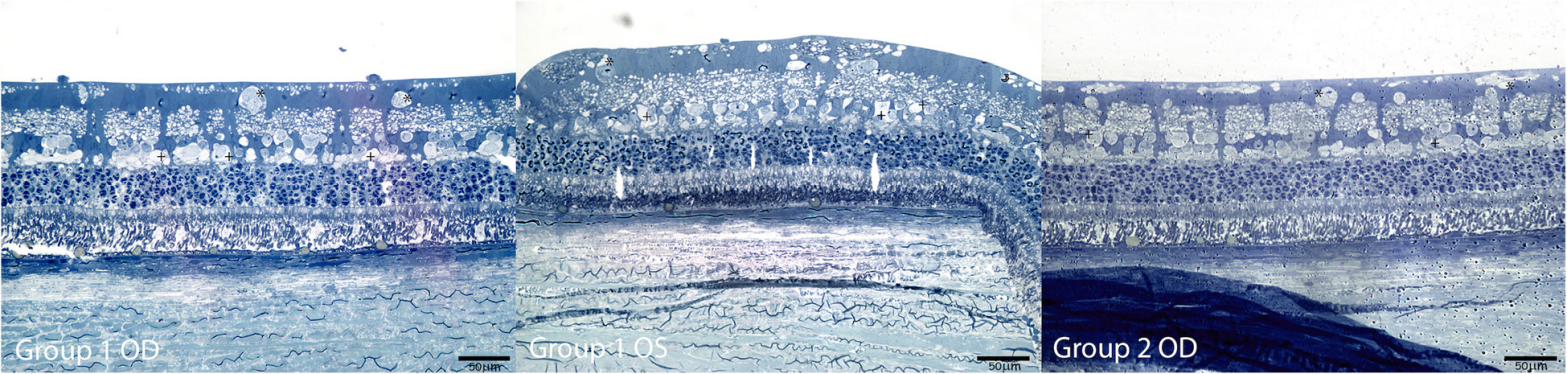
**Retinal findings by histology with 1% toluidine blue light microscopy 1 week after intravitreal 0.05 ml of Trisodium in OD and BSS in OS.** Group 1 OD: Ganglion cells (GC): focal areas of vacuolization (*); Inner plexiform layer (IPL): intact; inner nuclear layer (INL): focal vacuolization (+); outer plexiform layer (OPL): intact; Outer nuclear layer (ONL): artifactual findings; photoreceptors layer: artifactual findings. Group 1 OS: GC: focal areas of vacuolization (*); IPL: intact; INL: focal vacuolization (+); OPL: intact; ONL: artifactual findings; photoreceptors layer: artifactual findings. Group 2 OD: GC: focal areas of vacuolization (*); IPL: intact; INL: focal vacuolization (+); OPL: intact; ONL: artifactual findings; photoreceptors layer: artifactual findings.

**Figure 2:**
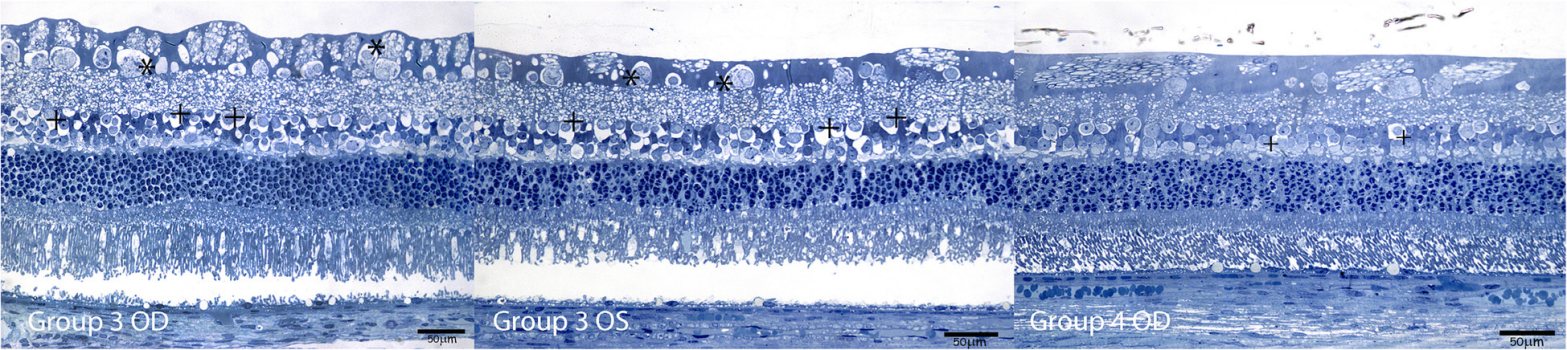
**Retinal findings by histology with 1% toluidine blue light microscopy 1 week after intravitreal 0.05 ml of Orangell in OD and BSS in OS.** Group 3 OD: GC: focal areas of vacuolization (*); IPL: intact; INL: focal vacuolization (+); OPL: intact; ONL: artifactual findings; photoreceptors layer: artifactual findings. Group 3 OS: GC: focal areas of vacuolization (*); IPL: intact; INL: focal vacuolization (+); OPL: intact; ONL: intact; photoreceptors layer: artifactual findings. Group 4 OD: GC: intact; IPL: intact; INL: focal vacuolization (+); OPL: intact; ONL: intact; photoreceptors layer: artifactual findings.

**Figure 3:**
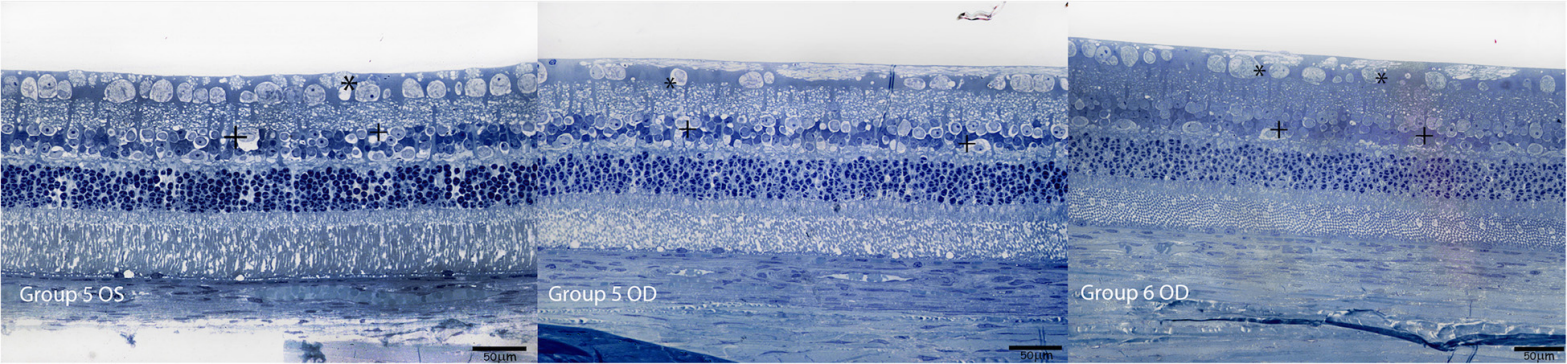
**Retinal findings by histology with 1% toluidine blue light microscopy 1 week after intravitreal 0.05 ml of Methyl Violet in OD and BSS in OS.** Group 5 OD: GC: focal areas of vacuolization (*); IPL: intact; INL: focal vacuolization (+); OPL: intact; ONL: artifactual findings; photoreceptors layer: artifactual findings. Group 5 OS: GC: focal areas of vacuolization (*); IPL: intact; INL: focal vacuolization (+); OPL: intact; ONL: intact; photoreceptors layer: artifactual findings. Group 6 OD: GC: focal areas of vacuolization (*); IPL: intact; INL: focal vacuolization (+); OPL: intact; ONL: intact; photoreceptors layer: intact.

No major anatomic signs of toxicity were found in the histology of both experimental and control eyes. The histopathological appearance of the retina, choroid and sclera was within “focal” abnormalities without any signs of severe retinal necrosis or cystic degeneration. Nerve fiber layer, retinal pigment epithelium (RPE) and choriocapillaris showed focal abnormalities after 1 week of the injection in every group analyzed. Concerning retinal damage, seven days after intravitreal dye injection, either the control or the study group examination in both concentrations showed only sparse regions in the retina of vacuolization and edema. Groups receiving Trisodium presented focal areas of vacuolization in the nerve fiber layer, ganglion cells layer and inner nuclear layer, with no major retinal damage (Figure 1). Groups receiving Orangell showed focal areas of vacuolization in the nerve fiber layer, ganglion cells layer, inner nuclear layer and outer nuclear layer, with no major retinal damage (Figure 2). Concerning groups receiving Methyl Violet, focal areas of vacuolization occurred in the nerve fiber layer, ganglion cells layer, inner nuclear layer and outer nuclear layer, with mild distortion of the retinal structure (Figure 3). One rabbit in Group 5 receiving Methyl Violet in the dose of 1.00 g/L presented focal areas of thinning of inner retina in both treated and control eyes and one rabbit in Group 6 receiving Methyl Violet in the dose of 0.50 g/L presented multinucleated cells in the ganglion cells layer.

### Functional retinal evaluation with ERG

At the doses tested, MV, TR and OR caused no statistical alterations in ERG during the follow-up period. Amplitude analysis of maximal scotopic b-wave showed no significant reduction in either dye injected or control eyes (p > 0.05). Scotopic b-wave amplitude in both eyes showed no statistical decrease (Table 2).

**Table 2:** **ERG scotopic maximal b-wave in baseline (pre max b-wave), endpoint (post max b-wave) amplitude description, and percentage comparison between them (% difference)**

	**Dye (OD)**		**Scotopic**			**BSS (OS)**		**Scotopic**	
	**Animal #**	**Pre max b-wave**	**Post max b-wave**	**% difference**		**Animal #**	**Pre max b-wave**	**Post max b-wave**	**% difference**
	#1	198,5	181,5	−8,6		#1	185	203,5	9,4
Group 1 - Trisodium 0,5 g/L	#2	145,5	144	−1	Group 1 - Trisodium 0,5 g/L	#2	155	120,5	−22,3
	#3	205,5	338	64,5		#3	219	150	−31,5
	#4	159	179,5	12,9		#4	161	151	−6,2
Group 2 - Trisodium 1,0 g/L	#5	151	215,5	42,7	Group 2 - Trisodium 1,0 g/L	#5	183	235,5	28,7
	#6	214	181	−15,4		#6	166	192,5	16
	#7	250	310	24		#7	182,5	261	43
Group 3 - Orangell(0,25 g/L)	#8	216,5	297	37,2	Group 3 - Orangell(0,25 g/L)	#8	176,5	223,5	26,6
	#9	154,5	183	18,4		#9	197	13,54	−17
	#10	100	192,5	92,5		#10	109,5	158	44,3
Group 4 - Orangell 1,0 g/L	#11	235	254	8,1	Group 4 - Orangell 1,0 g/L	#11	164	335,5	104,6
	#12	167	200	19,8		#12	108,5	175	61,3
	#13	263,5	205,5	−22		#13	216,5	332,5	53,6
Group 5 - Methyl Violet 1,0 g/L	#14	164	218	32,9	Group 5 - Methyl Violet 1,0 g/L	#14	198	206,5	4,3
	#15	235,5	228	−3,2		#15	210,5	140	−33,5
	#16	249,5	293,5	17,6		#16	313	258,5	−17,4
Group 6 - Methyl Violet 0,5 g/L	#17	228,5	243	6,3	Group 6 - Methyl Violet 0,5 g/L	#17	225,5	260,5	15,5
	#18	199	164	−17,6		#18	222	186	−16,2

A decrease in the post-injection response of >50% was considered remarkable.

## Discussion

Vital dyes may be used to facilitate intraoperative surgical procedures such as ILM or ERM-peeling [16-18]. In this rabbit-model study, the retinal biocompatibility of MV, TR and OR were determined to evaluate these dyes as alternatives in chromovitrectomy.

In the histological analysis of eyes injected with Trisodium, Orangell and Methyl Violet some aspects could be observed, such as slight distortion of major retina, none vitreous cells or alterations, focal areas of vacuolization in the nerve fiber layer and outer nuclear layer/photoreceptors with no other findings, except for one rabbit in Group 5 receiving Methyl Violet in the dose of 1.00 g/L that presented focal areas of thinning of inner retina in both treated and control eyes and one rabbit in Group 6 receiving Methyl Violet in the dose of 0.50 g/L that presented multinucleated cells in the ganglion cells layer. Overall, we observed herein good retinal biocompatibility of MV, TR and OR in our experiments in rabbits’ eyes.

The electroretinogram (ERG) was used for detecting and to quantify possible retinal toxicity after intravitreal dye injection in our experiments in rabbits’ eyes. Considering remarkable a decrease in the postinjection response of >50%, intravitreal MV, TR and OR injections caused no major changes in ERG-examination during the follow-up period. Nevertheless, normal ERG results do not rule out functional and possible structural changes at the level of ganglion cells or the nerve fiber layer. Maximal scotopic b-wave record was used as the most important parameter of toxicity according to other studies in literature supporting this to be the best evidence of drug toxicity in animal models [13,15].

The investigation of dyes and drugs applied on the retina may be accomplished in cell culture or animal experiments. Cell culture models with retinal pigment epithelial or neuroretinal cells, e.g., R28 cells, may allow systematic evaluation of drug toxicity in a controlled fashion. In a cell culture system, a chemical agent may be found to be safe at certain concentrations, while in-vivo evaluation may show specific signs of damage, such as vacuolization and loss of cells. These observations may result from the complexity of the intraocular milieu, making it difficult to replicate in a cell or tissue culture system.

Research in animal models is usually considered better evidence of pharmacological activity compared to cell culture alone, since it simulates better the human ocular and retinal environment. Results in human chromovitrectomy may not correspond to results obtained in animal models, as interspecies variations occur. In our research, we did not find considerable anatomical or functional damage to the retinal layers.

The limitation of our investigation is that only two concentrations were tested, in contrast to the large number of concentrations that can be examined in cell culture research. This study did not evaluate the affinity to the ILM; therefore it should be performed eventually. Also, this study does not rule out the possibility that longer-term follow-up in the New-Zealand albino rabbits or in human subjects might show untoward side effects. The comparison of potential and novel dye in an animal model is the main advantage of our study.

## Conclusion

In conclusion, Trisodium, Orangell and Methyl Violet did not induce significant ERG amplitude reduction or LM alterations in this preliminary experimental research. This suggests that the capacity of these three dyes to stain vitreous and preretinal tissues can be studied without major toxicity concerns and indicating that these dyes could potentially be used in future chromovitrectomy studies in order validate our findings.

## Abbreviations

BriB: Brilliant blue
BSS: Balanced salt solution
ERG: Electroretinography
ERM: Epiretinal membranes
FLI: Photopic 30 Hz flicker b- wave
ICG: Indocyanine green
ILM: Internal limiting membrane
LM: Light microscopy
MAX: Maximum scotopic a- wave
MV: Methyl violet
OD: Right eye
OR: Orangell
OS: Left eye
ROD: Minimal scotopic b-wave
RPE: Retinal pigment epithelium
SFC: Photopic single flash cone b-wave
TA: Triamcinolone acetonide
TB: Trypan blue
TR: Trisodium
